# Femtosecond laser-assisted laser in situ keratomileusis for the correction of high myopia in Meesmann corneal dystrophy: a case report

**DOI:** 10.5935/0004-2749.20230003

**Published:** 2022-01-31

**Authors:** Hassan Hashemi, Mohammadreza Aghamirsalim, Shiva Mehravaran, Soheila Asgari

**Affiliations:** 1 Noor Ophthalmology Research Center, Noor Eye Hospital, Tehran, Iran.; 2 Translational Ophthalmology Research Center, Tehran University of Medical Science, Tehran, Iran.; 3 ASCEND Center for Biomedical Research, Morgan State University Baltimore, MD, USA.

**Keywords:** Keratomileusis, laser in situ, Myopia, Corneal dystrophy, juvenile epithelial of Meesmann, Ceratomileuse assistida por excimer laser in situ, Miopia, Distrofia corneana epitelial juvenil de Meesmann

## Abstract

The patient was a 26-year-old woman who had manifest refraction and uncorrected and corrected distance visual acuities of -7.00 × -4.50 at 175°, 20/400, and 20/25, respectively, in the right eye, and -3.25 × -5.25 at 179°, 20/200, and 20/25, respectively, in the left eye. In the right and left eyes, the mean corneal thicknesses were 733 and 749 µm, and the maximum epithelial thicknesses were 70 and 68 µm, respectively. Myriads of intraepithelial cysts were observed in the slit-lamp examination. At 30 months after femtosecond laser-assisted laser in situ keratomileusis (femto-LASIK), the manifest refraction and uncorrected and corrected distance visual acuities were respectively 0.00 × -1.25 at 55°, 20/25, and 20/20 in right eye, and 0.00 × -0.50 at 135°, 20/20, and 20/20 in the left eye. In this case of Meesmann dystrophy, myopia was successfully treated with thick flap femto-LASIK without complications or ectasia.

## Abstract (pt)

A paciente era uma mulher de 26 anos com refração manifesta, acuidade visual para longe não corrigida e corrigida de -7,00 × -4,50 a175°, 20/400 e 20/25 no olho direito e -3,25 × -5,25 a 179°, 20/200 e 20/25 no olho esquerdo. A espessura média da córnea foi de 733 e 749 µm, e a espessura epitelial máxima foi de 70 e 68 µm, respectivamente no olho direito e no esquerdo. Inúmeros cistos intraepiteliais foram observados no exame com lâmpada de fenda. Trinta meses após o femto-LASIK, a refração manifesta, a acuidade visual para longe não corrigida e corrigida eram respectivamente de 0,00 × -1,25 a 55°, 20/25 e 20/20 no olho direito e 0,00 × -0,50 a 135°, 20/20 e 20/20 no olho esquerdo. Neste caso de distrofia de Meesmann, a miopia foi tratada com sucesso com femto-LASIK de retalho espesso sem complicações ou ectasia.

## INTRODUCTION

Meesmann corneal dystrophy is a rare *autosomal dominant* and bilateral disorder in which mutations in the KRT2 and KRT3 genes lead to a corneal dystrophy phenotype^(1)^. Slit-lamp findings include bubble-like punctate epithelium with transparent microcysts, which in some cases, can cause corneal irregularity, impaired vision, glare, photophobia, and patient discomfort^(2)^. In such cases, the treatment options include photorefractive keratectomy with mitomycin C (PRK-MMC)^(3)^ and keratoplasty^(4)^, and a previous study reported cases of postoperative recurrence^(3)^. We observed full recurrence of epithelial cysts in a case treated with PRK-MMC; therefore, the patient was treated with femtosecond laser-assisted keratomileusis (femto-LASIK) for the first time.

## CASE REPORT

The patient was a 26-year-old woman who was referred to our refractive surgery clinic because of contact lens intolerance and the desire for freedom from spectacles. Her father, who also had a case of Meesmann dystrophy, had complete disease recurrence 6 months after PRK-MMC along with severe loss of visual acuity. The patient’s medical history indicated no previous surgical intervention for correction of refractive errors, and only glasses and contact lenses were used. The corneal thickness readings in the right (oculus dexter [OD]) and left eyes (oculus sinister [OS]) were respectively 733 and 749 µm with an ultrasound pachymeter (Nidek UP-1000; Nidek Technologies, Gamagori, Japan), and 724 and 707 µm using Pentacam HR (Oculus Optikgeräte GmbH, Wetzlar, Germany). The endothelial cell density readings were 2646 and 3205 cells/mm^2^, respectively. In slit-lamp examination, myriads of intraepithelial cysts were clearly visible, and mild punctate epithelial erosions were observed in both eyes.

The Noor Eye Hospital Ethics Committee (ID No. M.1721) confirmed that approval was not required. The disease and treatment were fully explained to the patient, and a written informed consent was obtained. After topical anesthesia, 10.0-mm superior hinged flaps were created using Femto LDV Z6 (Ziemer Ophthalmic Systems AG, Port, Switzerland) with a thickness of 140 µm in the OD and 160 µm in the OS. The maximum epithelial thickness measured on RTVue-100 OCT (Optovue Inc., USA) was 70 µm in the OD and 68 µm in the OS. We chose a thicker flap to spare the cysts and the diseased cornea and to prevent post-LASIK inflammation from causing exacerbation and scarring. The corneal thickness was sufficient for this approach. After lifting the flap, stromal ablation was performed using the EX 500 laser platform set (WaveLight GmbH, Erlangen, Germany) for a 6.5-mm optical zone and a 1.25-mm blend zone. In the OD and OS, the target refractions were +0.50 × 0.00 at 175° and +0.50 × 0.00 at 180°, the ablation depths were 164.73 and 126.52 µm, and the residual stromal bed (RSB) thicknesses were 428.27 and 462.48 µm, respectively.

The postoperative treatment included chloramphenicol 0.5% eye drops (Sina Darou, Tehran, Iran) every 6 hours for 3 days and betamethasone 0.1% (Sina Darou) every 6 hours for 7 days. No intraoperative complications were encountered. The postoperative examination on the third day revealed dry eyes, predominantly in the right eye; this was treated with preservative-free artificial tears every 6 hours for 1 month.

**Figure 1.**
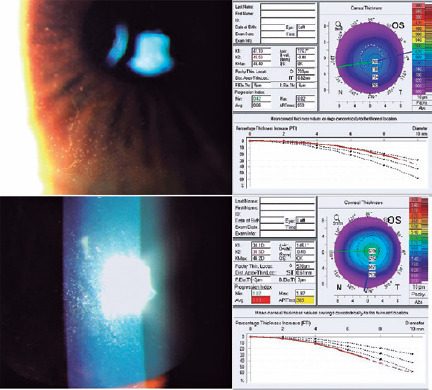
Slit-lamp photographs illustrating the epithelium (left) and pachymetry maps (right) of the left eye in the case of Meesmann corneal dystrophy before (above) and 30 months (below) after femtosecond laser-assisted keratomileusis.

The 30-month outcomes in terms of vision and refraction are summarized in table 1. At baseline and 12, 24, and 30 months after surgery, corneal astigmatism decreased from 3.5 at 177.5° to 1.4 at 70.7°, 0.9 at 54.2°, and 0.8 at 63.0° in the OD, and from 4.4 at 176.7° to 0.3 at 132.1°, 0.5 at 170.5°, and 0.4 at 145.1° in the OS. The topographic pachymetry changed from 724 µm to 549, 561, and 570 µm in the OD and from 707 µm to 578, 569, and 578 µm in the OS. At 30 months, the maximum keratometry had changed from 45.7 at baseline to 46.0D in the OD and 46.4-46.2D in the OS. No sign of ectasia was found in the topographic or ophthalmic examinations. The minimum RSB measured on RTVue-100 OCT was 510 µm in the OD and 539 µm in the OS, indicating no changes as compared with the baseline values. Moreover, slit-lamp examination revealed no scars or exacerbation of dystrophy (Figure 1). The patient satisfaction score was 9/10 in the OD and 10/10 in the OS.

**Table 1.** Thirty-month changes in vision and manifest refraction in a myopic case of Meesmann dystrophy

		Baseline	1 month	6 months	12 months	24 months	30 months
UDVA	OD	20/400	20/25	20/25	20/30	20/25	20/25
	OS	20/200	20/25	20/30	20/20	20/20	20/20
CDVA	OD	20/25	20/20	20/20	20/20	20/20	20/20
	OS	20/25	20/20	20/20	20/20	20/20	20/20
Refraction (D)	OD	-7.00 × -4.50 at 175°	-0.75 × -0.25 at 119°	+1.00 × -1.50 at 40°	+1.2 × -1.50 at 50°	+0.5 × -1.25 at 55°	0.00 × -1.25 at 55°
	OS	-3.25 × -5.25 at 179°	+0.25 × -1.50 at 143°	0.00 × -0.50 at 110°	0.00 × -0.50 at 130°	-0.25 × -0.5 at 120°	0.00 × -0.5 at 135°

UDVA= uncorrected distance visual acuity; CDVA= corrected distance visual acuity; D= diopter.

## DISCUSSION

Meesmann corneal dystrophy is a disorder of the epithelium and basement membrane. Although cysts are intraepithelial, superficial keratectomy procedures such as lamellar or penetrating keratoplasty as treatment options have not been successful^(5)^. The second option is PRK-MMC, in which the removal of the epithelium and photoablation of the underlying layers could reduce flare-ups. However, this option had poor results, including recurrence, exacerbated symptoms, corneal scarring, and impaired vision^(3, 4, 6)^. As our experience and the literature indicated that any intervention that involves cyst manipulation can have adverse outcomes and reduced vision, in this case, we decided to correct the refractive errors using femto-LASIK. Thirty-month follow-up results confirmed that femto-LASIK with a thick flap allowed correction of the myopia and improved vision by avoiding the cyst areas. At 30 months after surgery, the UDVAs in the OD and OS were improved by 8 and 9 Snellen lines, respectively, with one stable line of improvement in the CVDA bilaterally. Residual astigmatism was found in the right eye, which could be attributed to the higher degree of myopia in the right eye than in the left eye^(7, 8)^.

In a case of mild myopia treated with PRK-MMC, Ghanem et al.^(3)^ reported decreases in visual acuity of 3 and 1 Snellen line due to residual refractive error. Moreover, relative recurrence of tiny microcysts at 3 months and complete recurrence at 1 year after surgery were observed. In another case reported by Yeung et al.^(6)^, the OD lost 3 lines of CDVA by 3 months after surgery, which persisted after retreatment; a 1-line increase was observed after the third PRK-MMC as compared with baseline. In the OS, the preoperative CDVA declined from 20/30 to 20/70 postoperatively. Despite the use of MMC, which is effective for treating epithelial tumors, PRK has failed to achieve stable results and can be associated with vision loss. It is also unable to prevent the recurrence of Meesmann corneal dystrophy.

In conclusion, in the case presented herein, a stable improvement in vision was achieved without ectasia, complications, or exacerbation of dystrophy at 30 months after femto-LASIK treatment for myopia. In addition, the patient was completely satisfied with the treatment. Another option that we can suggest is femto-SMILE (small-incision lenticule extraction) but still needs to be tested.
